# 17β-estradiol preserves right ventricular function in rats with pulmonary arterial hypertension: an echocardiographic and histochemical study

**DOI:** 10.1007/s10554-018-1468-0

**Published:** 2018-10-22

**Authors:** Yi-Dan Wang, Yi-Dan Li, Xue-Yan Ding, Xiao-Peng Wu, Cheng Li, Di-Chen Guo, Yan-Ping Shi, Xiu-Zhang Lu

**Affiliations:** 1 Department of Echocardiography, Heart Center, Beijing Chao Yang Hospital, Capital Medical University, 8 Gongren Tiyuchang Nanlu, Chaoyang District, Beijing, 100020 China; 2 Beijing Key Laboratory of Hypertension, Beijing, 100020 China

**Keywords:** Sex difference, Pulmonary arterial hypertension, Estradiol, Echocardiography, Ventricular function

## Abstract

Pulmonary arterial hypertension (PAH) is more prevalent in females. Paradoxically, female patients have better right ventricular (RV) function and higher survival rates than males. However, the effects of 17β-estradiol (E2) on RV function in PAH has not been studied. Twenty-four male rats were exposed to monocrotaline (MCT) to induce experimental PAH, while treated with E2 or vehicle respectively. Together with eight control rats, thirty-two rats were examined by echocardiography 4 weeks after drug administration. Echocardiographic measurement of RV function included: tricuspid annular plane systolic excursion (TAPSE), RV index of myocardial performance (RIMP), RV fractional area change (RVFAC) and tricuspid annular systolic velocity (s′). RV free wall longitudinal strain (RVLS_FW_) and RV longitudinal shortening fraction (RVLSF) were also used to quantify RV function. RV morphology was determined by echocardiographic and histological analysis. TAPSE, RVFAC and s′ were reduced, and RIMP was elevated in the MCT-treated group and vehicle-treated group, when compared with control group (*P* < 0.01). TAPSE, RVFAC and s′ in the E2 group were higher, while RIMP was lower than those in the MCT-treated group and vehicle-treated group (*P* < 0.01). Myocardial functional parameters (RVLS_FW_ and RVLSF) were also higher in the E2 group. Enhanced serum E2 levels were closely correlated with the improvement in RV functional parameters and enhancement of serum BNP levels (*P* < 0.01 for all groups). RV function decreased significantly in male rats with MCT-induced PAH, while E2 exhibited a protective effect on RV function, suggesting that E2 is a critical modulator of sex differences in PAH.

**Electronic supplementary material:**

The online version of this article (10.1007/s10554-018-1468-0) contains supplementary material, which is available to authorized users.

## Introduction

Pulmonary arterial hypertension (PAH; WHO group 1 pulmonary hypertension) is an idiopathic chronic lung disease characterized by pulmonary vascular remodeling and a progressive increase in pulmonary artery pressure, leading to right ventricle (RV) failure and death [1].

It has been reported that gender differences exist in both experimental animals and patients with PAH. Despite females having higher prevalence and incidence rates of PAH [2–4], women with PAH exhibit better survival than men [3–5]. This incongruous finding is termed the “estrogen paradox in PAH” [6]. Interestingly, female PAH patients have a higher RV ejection fraction (RVEF) than males at baseline [7, 8] and a better RVEF response after medical treatment [9] than their male counterparts. Recent studies also demonstrated that the preserved RVEF in women is a major contributor to the female survival advantage in PAH [9]. The differences in RV function are at least partly mediated by the effects of sex hormones, as evidenced by studies which demonstrated higher estradiol levels are associated with better RV systolic function in postmenopausal women on hormone replacement therapy [10]. RV function is recognized as an important prognostic determinant in PAH [11]. Besides focusing on the effects of estrogen on pulmonary vascular remodeling, more and more studies have started to investigate the effects of 17β-estradiol (E2) on RV remodeling and function, mainly regarding hemodynamic changes and mechanisms [12, 13]. Transthoracic echocardiography has been widely used as a noninvasive method for the evaluation of RV function in PAH [14]. Conventional parameters for the measurement of RV function include tricuspid annular plane systolic excursion (TAPSE), RV fractional area change (RVFAC), RV index of myocardial performance (RIMP), and tricuspid annular systolic velocity (s′). Recently, the measurement of RV free wall longitudinal strain (RVLS_FW_) by two-dimensional speckle tracking imaging (2D-STI) has become a standard measurement, which can quantify complex cardiac motions independent of angle. Moreover, RV longitudinal shortening fraction (RVLSF) measured by 2D-STI, another parameter to evaluate RV function, was shown to be well correlated with RVEF measured by cardiac magnetic resonance (CMR) [15].

The aim of this study was to determine whether E2 exerts beneficial effects on RV function and RV remodeling in a rodent model of PAH. We evaluated RV function comprehensively by echocardiography and tried to explain the sex differences in PAH from echocardiographic and histochemical perspectives.

## Methods

### Ethic statement

All animal experiments were in compliance with the National Institutes of Health Guide for the Care and Use of Laboratory Animals and were approved by the Animal Care and Use committee of Capital Medical University.

### Animals and treatment

Male 8 week old Sprague–Dawley rats (300–350 g) were used in this study. Animals had access to food and water *ad libitum* during the experimental period. To induce PAH, rats were treated with a single subcutaneous injection of monocrotaline (MCT) (60 mg/kg, Sigma). This model of PAH is widely used and has been shown to be efficient and reproducible [16–18]. The rats were randomly assigned to the following treatment groups: (1) MCT-treated group (n = 8): MCT without any other treatment; (2) MCT + E2 group (n = 8): MCT-exposed male rats received E2 (75 µg/kg/d, sigma) via subcutaneous osmotic minipumps (model 2ML4; Alzet, Cupertino, CA, USA) for 1 week before, and for the entire 4-week after MCT treatment; (3) MCT + vehicle group (100% ethanol, n = 8): vehicle was given at the same time as E2 group after MCT treatment; and (4) control group (n = 8). The E2 administration resulted in physiological E2 levels for adult female Sprague–Dawley rats, as reported in other studies [19, 20]^,^ and confirmed by the serum level of E2 measured in this study.

### Echocardiographic examinations

Rats were anesthetized intraperitoneally with 10% chloral hydrate. Images were obtained by using a Philips EPIQ 7C (Philips Healthcare, MA, USA) equipped with 4−12 MHz probe. Heart rate fluctuated between 350 and 430 beats per minute during the examination. Images of the apical 4-chamber view (AP4) and parasternal short-axis view (PSAX) were recorded with a high frame rate (197–205 frames per second). Additional details can be found in the Data Supplement.

### Right heart catheterization

After echocardiographic assessment, all rats underwent right heart catheterization for hemodynamic evaluation. The pulmonary pressures were measured by the closed-chest technique reported in other studies [21]. Briefly, a 13-cm-long, heparin-priming polyethylene catheter (outer diameter, 0.9 mm), connected to PowerLab 16/30 (ADInstruments, Dunedin, New Zealand) through a pressure transducer, was introduced into the right internal jugular vein and advanced into the RV and further into the main pulmonary artery. The right atrial pressure (RAP), RV systolic pressure (RVSP), and mean pulmonary arterial pressure (mPAP) were recorded. Hemodynamic values were automatically calculated by the physiological data acquisition system (LabChart 7 Software; ADInstruments Co., Shanghai, China).

### Assessment of serum E2, BNP and right ventricular hypertrophy

Blood samples were collected from the inferior vena cava immediately after right heart catheterization and allowed to stand for two hours before being centrifuged for 10 min at 3000 rpm. The supernatant serum was collected and stored at − 80 °C. Levels of serum E2 and BNP were determined by commercial enzyme-linked immunosorbent assay (ELISA) kit (E2: mouse/rat E2 ELISA kit, Calbiotech, Spring Valley, CO; BNP: Raybiotech, Norcross, GA, USA), following the manufacturer’s protocol.

RV hypertrophy (RVH) was assessed by Fulton index method as described previously [20, 22]. Briefly, after the isolation of the heart, two atria and the major vessels were removed from the ventricles. The RV was dissected away from the left ventricle (LV) and interventricular septum (LV + IVS), and weighed. The degree of RVH was expressed as the ratio of RV to (LV + IVS). The ratio of RV weight to body weight (RV/BW) was also calculated for assessment of RVH.

### Immunohistochemistry

The extent of RV fibrosis was determined by immunohistochemical staining. Additional details can be found in the Data Supplement.

### Statistical analysis

Continuous variables Were expressed as mean ± SEM. Experimental groups were compared by one-way analysis of variance (ANOVA) followed by Turkey’s post hoc test. Linear regression analysis was used to assess the association between two variables. The inter-observer agreement and intra-observer reproducibility was assessed using the Bland–Altman method. *P* < 0.05 was considered to be statistically significant. SPSS (version 17.0 for Windows; SPSS Inc., Chicago, IL, USA) was used for statistical analysis.

## Results

### Animal basic characteristics and E2’s effects on mortality and hemodynamics

Male Sprague–Dawley rats were divided into four experimental groups as described in the Methods. Except for the control group, the other three groups were injected with a single dose of MCT to induce PAH. After MCT injection, the relative amount of weight gain decreased dramatically, and was more obvious in the E2 treatment group, consistent with previous studies [23] (Table 1).

**Table 1:** Body weight, right ventricular morphological, hemodynamic and serum parameters

	Control	MCT	MCT + E2	MCT + vehicle	*P* value
Weights					
Baseline weight, g	325.83 ± 5.17	338.33 ± 3.73	330.00 ± 2.84	324.17 ± 1.89	0.070
Terminal weight, g	495.83 ± 16.11	432.67 ± 6.18^*^	369.83 ± 14.60^†‡^	427.20 ± 10.37^†^	< 0.001
Weight gain,%	51.83 ± 2.89	28.00 ± 2.33^†^	12.00 ± 0.04^†§^	31.40 ± 3.20^†^	< 0.001
RV/BW, mg/g	0.44 ± 0.16	1.41 ± 0.09^†^	0.73 ± 0.05^§^	1.33 ± 0.11^†^	< 0.001
RV/LV + IVS	0.26 ± 0.01	0.61 ± 0.02^†^	0.36 ± 0.01^§^	0.63 ± 0.05^†^	< 0.001
Hemodynamics					
RVSP, mmHg	25.22 ± 0.85	63.58 ± 2.97^†^	56.38 ± 1.05^†^	62.46 ± 4.82^†^	< 0.001
mPAP, mmHg	15.77 ± 0.52	35.29 ± 1.68^†^	31.72 ± 0.51^†^	36.78 ± 2.36^†^	< 0.001
RAP, mmHg	4.93 ± 1.18	10.64 ± 0.28^†^	10.36 ± 0.30^†^	11.32 ± 1.68^†^	< 0.001
Serum parameters					
E2 (pg/ml)	5.55 ± 0.59	7.80 ± 0.68	14.07 ± 1.45^†§^	8.09 ± 0.62	< 0.001
BNP (pg/ml)	161.33 ± 15.04	727.33 ± 43.22^†^	260.00 ± 18.22	759.40 ± 44.12^†^	< 0.001

Data are presented as means ± SEM. N = 5–8 per group

*BSA* body surface area, *RV* right ventricle, *BW* body weight, *LV* left ventricle, *IVS* interventricular septum, *RVSP* right ventricular systolic pressure, *mPAP* mean pulmonary arterial pressure, *RAP* right atrial pressure, *E2* 17β-estradiol, *BNP* B-type natriuretic peptide

*P < 0.05 when compared with the control group

^†^*P* < 0.01 when compared with the control group

^‡^*P* < 0.05 when compared with the MCT-treated group

^§^*P* < 0.01 when compared with the MCT-treated group

In a previous study, the administration of E2 for 2 weeks starting from day 21 after MCT injection improved survival of PAH rats [18]. In our study, three rats from the MCT-treated group and two rats from the vehicle-treated group died during the 4-week experimental period, while none died in the E2-treated group. Regarding the hemodynamic parameters, although RVSP and mPAP were slightly lower in the E2-treated group compared to the MCT-exposed group, the RV pressure overload showed no significant difference between the two groups (RVSP and mPAP, *P* = 0.065). RAP also showed no difference between the two groups (RAP, *P* = 0.486) (Table 1).

### E2 lessens RV morphologic changes in MCT-induced PAH rats

MCT induced increased pulmonary arterial pressure leading to morphologic changes in the RV. In this study, we used both 2D echocardiography, as well as gross and histological measurements to evaluate the morphologic changes in the RV. The increases in RV diameter, RVD/LVD and eccentricity index (EI) measured by echocardiography, indicated RV enlargement. Both the increase in RV wall thickness and the increase in RV weight, expressed as RV/LV + IVS and RV/BW, indicated the presence of RVH. Regarding histological measurements, RVH was also identified by the measurement of mean cross sectional area (CSA) of RV cardiomyocytes. We also evaluated the extent of RV fibrosis to evaluate RV myocardial remodeling.

### E2 attenuates MCT-induced RV enlargement

RV diameter, including RV basal diameter and RV mid diameter, increased dramatically in the MCT-exposed group compared with the control group (*P* < 0.01). This was especially the case for the RV mid diameter, which showed a more significant increase compared with the basal portion (Table 2). E2 treatment maintained the RV at a much smaller size compared with that of the MCT-exposed group (*P* < 0.01). RVD/LVD and EI also increased remarkably in the diseased groups without E2 treatment (*P* < 0.01) (Table 2).

**Table 2:** Right heart morphological and functional measurement by echocardiography

	Control	MCT	MCT + E2	MCT + vehicle	*P* value
	(n = 8)	(n = 5)	(n = 8)	(n = 6)	
RV morphology					
RV basal diameter, mm	3.88 ± 0.10	6.29 ± 0.23^†^	4.42 ± 0.21^§^	6.20 ± 0.23^†^	< 0.001
RV mid diameter, mm	3.38 ± 0.16	8.14 ± 0.34^†^	3.80 ± 0.26^§^	7.64 ± 0.27^†^	< 0.001
RVD/LVD	0.71 ± 0.02	1.36 ± 0.08^†^	0.88 ± 0.06^§^	1.28 ± 0.06^†^	< 0.001
RV wall thickness, mm	0.82 ± 0.05	2.02 ± 0.09^†^	0.98 ± 0.04^§^	2.04 ± 0.05^†^	< 0.001
EI	0.91 ± 0.07	1.57 ± 0.04^†^	0.95 ± 0.04^§^	1.50 ± 0.06^†^	< 0.001
RV EDA indexed to BW (cm^2^/kg)	0.74 ± 0.04	1.66 ± 0.07†	1.01 ± 0.11§	1.59 ± 0.09†	< 0.001
RV ESA indexed to BW (cm^2^/kg)	0.40 ± 0.03	1.34 ± 0.07†	0.62 ± 0.09§	1.28 ± 0.06†	< 0.001
RA area indexed to BW (cm^2^/kg)	0.35 ± 0.04	1.10 ± 0.06^†^	0.58 ± 0.06^§^	1.08 ± 0.07^†^	< 0.001
RV function					
Hemodynamic parameters					
VTI, cm	7.45 ± 0.43	2.61 ± 0.20^†^	5.97 ± 0.20^*§^	2.76 ± 0.32^†^	< 0.001
CI, mL/min/kg	202.70 ± 19.29	136.71 ± 9.27^*^	247.36 ± 13.24^§^	130.98 ± 14.78^*^	< 0.001
Conventional parameters					
TAPSE, mm	3.11 ± 0.08	1.01 ± 0.07^†^	2.69 ± 0.17^§^	1.07 ± 0.07^†^	< 0.001
RVFAC, %	46.17 ± 1.71	21.73 ± 1.68^†^	38.95 ± 1.96^§^	23.18 ± 2.32^†^	< 0.001
RIMP	0.36 ± 0.08	0.87 ± 0.08^†^	0.46 ± 0.06^§^	0.84 ± 0.07^†^	< 0.001
s′	6.85 ± 0.18	3.91 ± 0.25^†^	6.04 ± 0.35^§^	3.96 ± 0.23^†^	< 0.001
STI parameters					
RVLS_FW_ (%)					
Global	− 39.28 ± 1.13	− 29.52 ± 1.61^†^	− 37.40 ± 1.55^§^	− 27.98 ± 1.64^†^	< 0.001
Basal	− 40.67 ± 1.11	− 30.50 ± 1.41^*^	− 37.50 ± 2.63	− 29.00 ± 2.80^†^	0.002
Mid	− 35.83 ± 1.31	− 26.83 ± 2.31^*^	− 35.00 ± 2.11^‡^	− 27.67 ± 1.17^*^	0.002
Apical	− 42.50 ± 2.36	− 30.67 ± 1.41^†^	− 40.17 ± 2.30^‡^	− 28.17 ± 1.82^†^	< 0.001
RV LSF (%)	15.18 ± 1.33	9.17 ± 0.80^†^	14.28 ± 1.11^§^	9.25 ± 0.63^†^	< 0.001

Data are presented as means ± SEM

*PAH* pulmonary arterial hypertension, *RV* right ventricle, *RVOT* RV outflow tract, *RVD* right ventricular diameter, *LVD* left ventricular diameter, *EI* eccentricity index, *EDA* end-diastolic area, *ESA* end-systolic area, *BW* body weight, *RA* right atrium, *VTI* velocity time integral, *CI* cardiac index, *TAPSE* tricuspid annular plane systolic excursion, *RVFAC* RV fractional area change, *RIMP* right ventricular index of myocardial performance, *s′* tissue Doppler-derived tricuspid lateral annular systolic velocity, STI speckle tracking imaging, *RVLS*_*FW*_ RV free wall longitudinal strain, RV*LSF* RV longitudinal shortening fraction

*P < 0.05 when compared with the control group

^†^*P* < 0.01 when compared with the control group

^‡^*P* < 0.05 when compared with the MCT-treated group

^§^*P* < 0.01 when compared with the MCT-treated group

### E2 prevents MCT-induced RVH

**Fig. 1:**
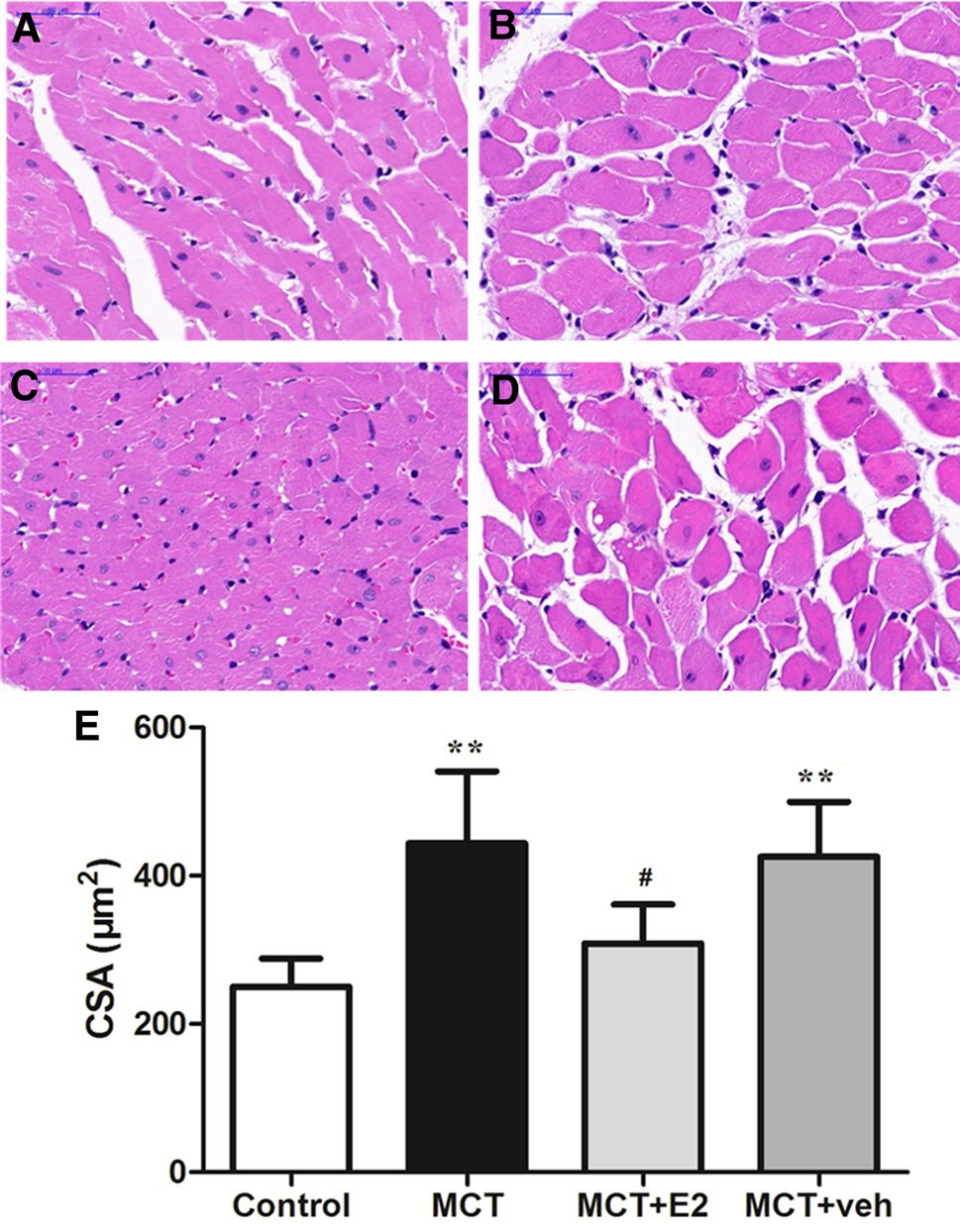
E2 administration prevented RV cardiomyocyte hypertrophy in rats with MCT-induced PAH. Representative photomicrographs of cross-sections with HE staining for RV cardiomyocytes from the following experimental groups: **a** control group; **b** MCT-treated group; **c** MCT + E2 group; and **d** MCT + vehicle group. Original magnification, × 400, scale bar = 50 µm. **e** Quantitative analysis of mean cross sectional area (CSA) of RV cardiomyocytes in rats. Values are expressed as mean ± SEM. **P < 0.01 versus the Control group, ^#^*P* < 0.05 versus the MCT-treated PAH group (one-way ANOVA with post hoc Turkey’s test). N = 5−8 per group. *MCT* monocrotaline; E2, 17β-estradiol

RV wall thickness was significantly higher in the MCT-injected rats compared with rats without MCT injection (*P* < 0.01) (Table 2), as was RV/LV + IVS and RV/BW (*P* < 0.01) (Table 1). HE staining of the RV showed that the CSA of cardiomyocytes in the MCT-treated group was significantly greater than that of the control group (*P* < 0.01). Treatment with E2 prevented the increase in RV wall thickness measured by echocardiography, as well as the increase in RV/LV + IVS, RV/BW ratios (*P* < 0.01 for all groups) (Table 1) and the CSA of cardiomyocytes (*P* < 0.05) (Fig. 1).

### E2 reduces the extent of RV fibrosis induced by MCT

**Fig. 2:**
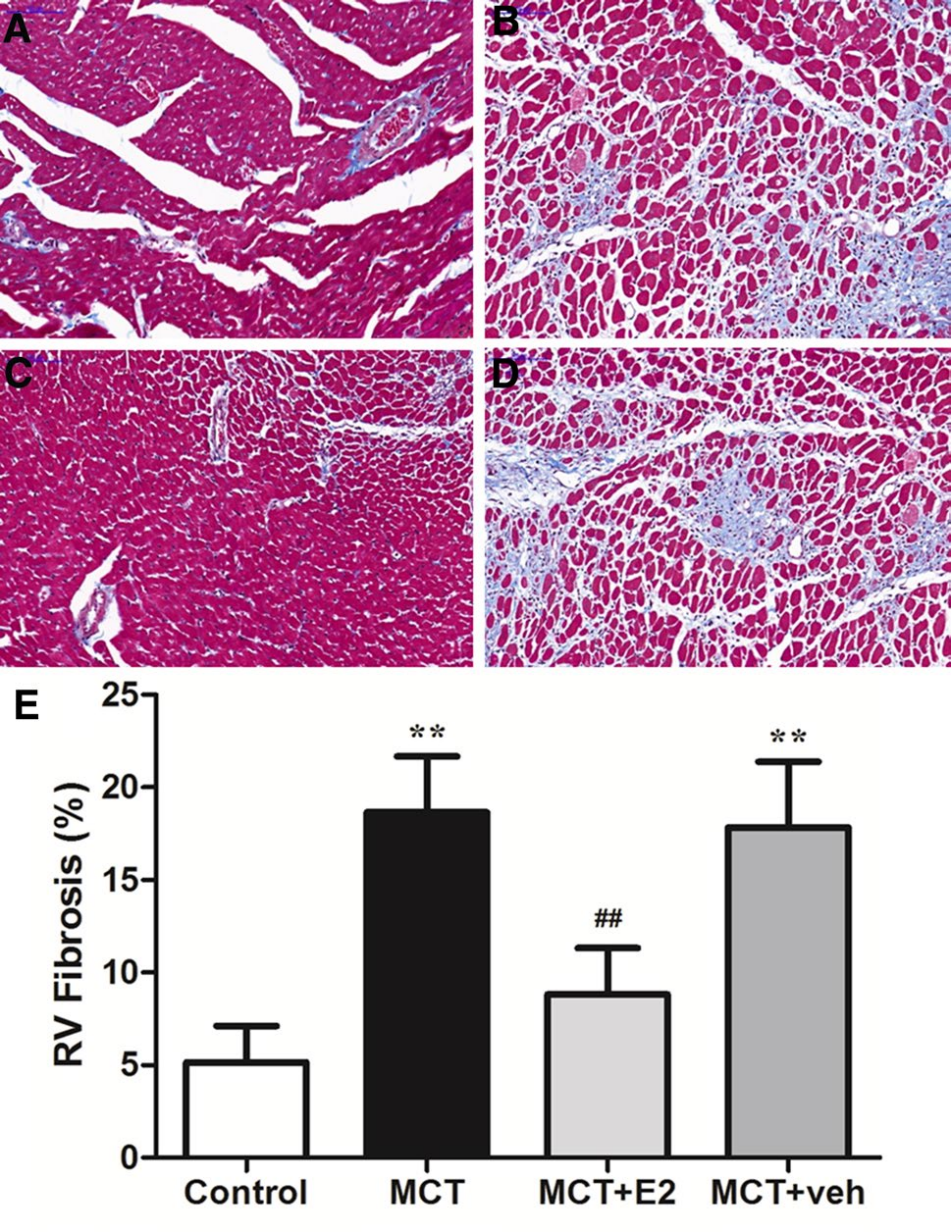
E2 administration reduced RV fibrosis in rats with MCT-induced PAH. Representative Masson’s Trichrome staining of RV free wall cardiomyocyte sections showing the extent of RV fibrosis (blue-stained areas) for the following experimental groups: **a** control group; **b** MCT-treated group; **c** MCT + E2 group; **d** MCT + vehicle group. Original magnification, × 200. **e** Quantitative analysis of RV fibrosis in cardiomyocyte sections (blue-stained areas expressed as percentage of total RV surface area). Values are expressed as mean ± SEM. **P < 0.01 versus the control group, ^##^*P* < 0.01 versus the MCT-treated PAH group (one-way ANOVA with post hoc Turkey’s test). N = 5−8 per group. *MCT* monocrotaline; E2, 17β-estradiol

RV remodeling is partly due to the increase of interstitial fibrosis, which manifested as the increase of myocardial collagen content. As demonstrated by Masson’s Trichome staining, significant RV fibrosis was observed in RV tissue in MCT-induced PAH rats, while E2 treatment dramatically attenuated the extent of RV fibrosis (Fig. 2).

### E2 improves RV function in MCT-induced PAH rats

The evaluation of RV function by echocardiography included traditional RV function parameters and STI parameters, as well as hemodynamic parameters.

### Conventional parameters of RV function

**Fig. 3:**
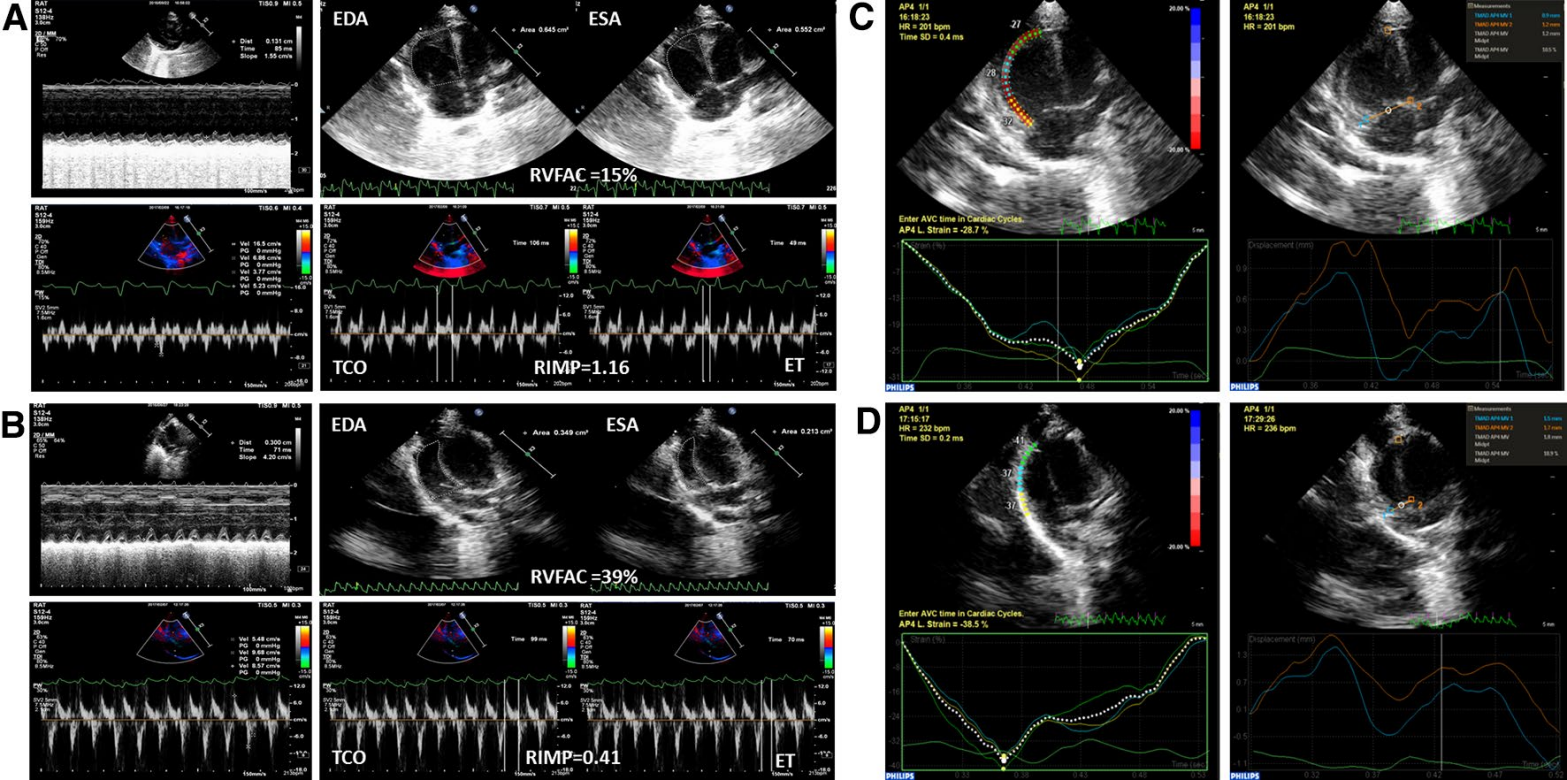
The evaluation of RV function in MCT-induced PAH rats and with E2 treatment by conventional echocardiography and STI. **a** Conventional parameters of RV function in PAH rats: TAPSE (upper left panel), RVFAC (upper right panel), s′ (lower left panel) and RIMP (lower right panel); **b** conventional parameters of RV function in PAH rats with E2 treatment: TAPSE (upper left panel), RVFAC (upper right panel), s′ (lower left panel) and RIMP (lower right panel); **c** STI analysis of PH rats: RVLS_FW_ (upper left panel) and RVLSF (upper right panel); and **d** STI analysis of PAH rats with E2 treatment: RVLS_FW_ (lower left panel) and RVLSF (lower right panel). *EDA* end-diastolic area, *ESA* end-diastolic area, *TCO* tricuspid closure-open Time, *ET* ejection time

With regard to traditional parameters, MCT-exposed rats had a lower TAPSE, RVFAC and s′, and higher RIMP compared to rats without MCT injection (*P* < 0.01 for all groups). Compared with the MCT-treated group, significant improvement of TAPSE, RVFAC, RIMP and s′ were observed in the E2 treatment group (*P* < 0.01 for all groups), which maintained RV function in MCT-treated rats similar to the control rats (Table 2; Fig. 3a, b).

### STI parameters of RV function

STI analyses demonstrated similar changes in RV function in rats from the E2-treated group. Strain analysis showed a significant increase in RVLS_FW_ after E2 treatment (*P* < 0.01), indicating that E2 improved RV myocardial function even under highly increased pressure overload. While, the strain of the basal segment showed no significant difference between the MCT-exposed group and the E2 group; however, the strain of the mid and apical segments was increased significantly following E2 treatment. RVLSF measured by STI also showed a dramatic increase in rats from the E2 treatment group (*P* < 0.01) (Table 2; Fig. 3c, d).

### Hemodynamic parameters

CI, is a hemodynamic parameter measured by echocardiography and reflects global RV function. In our study, CI was increased in MCT-injected rats with E2 treatment (Table 2). In terms of weight loss after E2 treatment, CI in the E2-treated group was even higher than the MCT-treated group. Coinciding with CI, VTI also showed an increase in the E2 group (Table 2).

### E2 suppresses the increase in serum BNP levels

BNP is a marker of RVH in PAH and its level is correlated with the degree of RV remodeling [24]. Our results showed that the serum BNP levels were markedly higher in rats from the MCT-induced PAH group than in the E2-treated group (Table 1), suggesting that RV dysfunction was significantly suppressed by E2 treatment.

### Correlation analysis of E2 levels and RV morphologic, functional and hemodynamic parameters

The serum estrogen levels were remarkably positively correlated with conventional RV functional parameters including TAPSE (r = 0.845, *P* < 0.001), RVFAC (r = 0.859, *P* < 0.001) and s′ (r = 0.802, *P* < 0.001), and was negatively correlated with RIMP (r = −0.803, *P* < 0.001). E2 levels were also positively correlated with STI parameters: RVLS_FW_ (r = 0.619, *P* = 0.006) and RVLSF (r = 0.637, *P* = 0.004).

According to the correlation analysis, E2 levels were inversely correlated with RV enlargement (EI: r = −0.774, *P* < 0.001; RVD/LVD: r = −0.745, *P* < 0.001) and RVH (RV/BW: r = −0.721, *P* < 0.001; RV/LV + IVS: r = −0.675, *P* = 0.002).

Regarding hemodynamic parameters, E2 levels was positively correlated with CI measured by echocardiography (r = 0.619, *P* = 0.008). However, there was no significant correlation between E2 levels and RVSP, mPAP and RAP evaluated by RHC (RVSP: r = −0.321, *P* = 0.195; mPAP: r = −0.400, *P* = 0.100; RAP = r = −0.196, *P* = 0.435). A remarkable negative correlation between the serum levels of E2 and BNP was demonstrated (r = −0.720, *P* = 0.001).

### Reproducibility

**Fig. 4:**
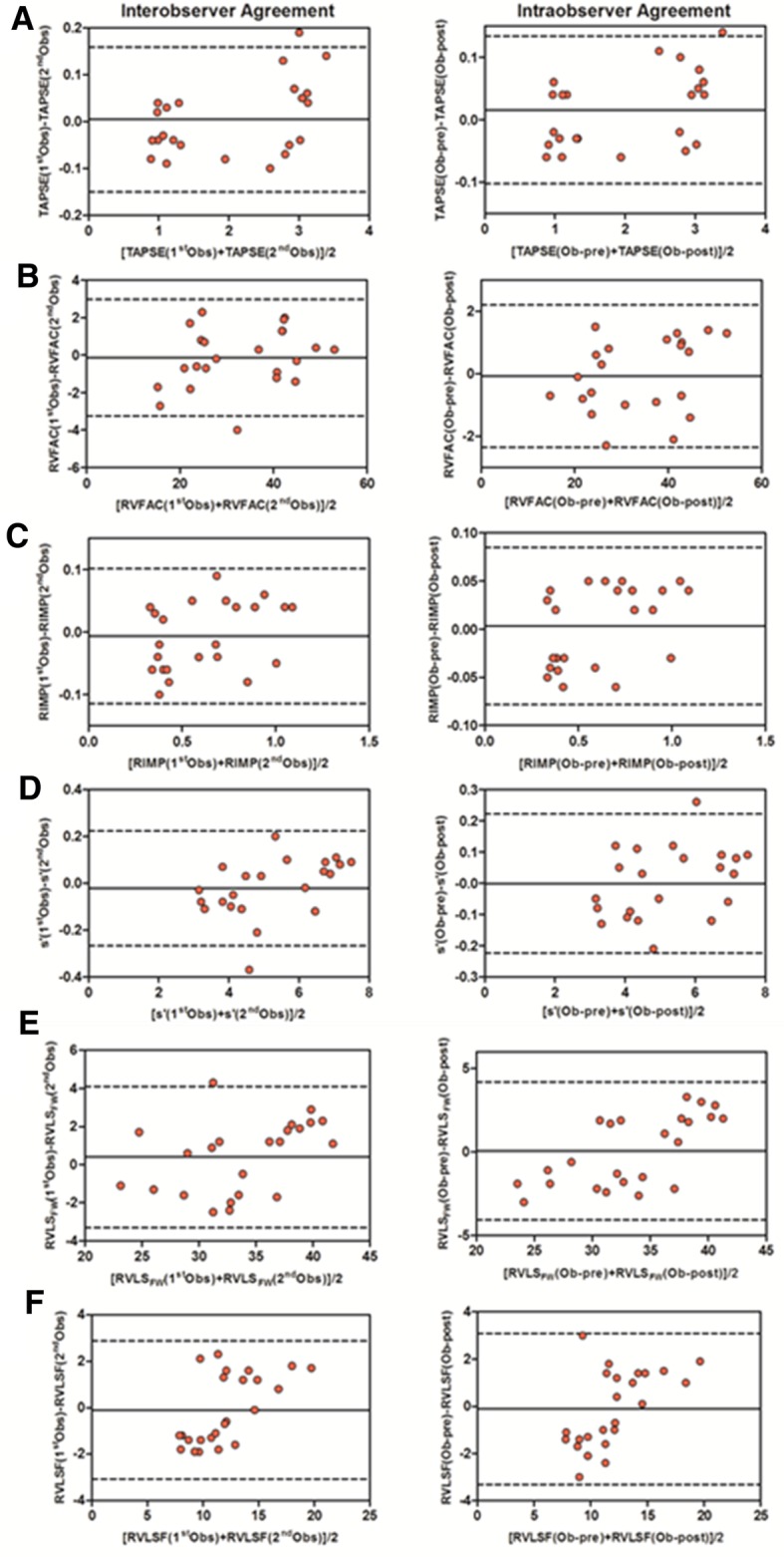
The assessment of inter-observer agreement and intra-observer reproducibility. By using the Bland–Altman method, all the data presented were evaluated for inter-observer agreement and intra-observer reproducibility through the comparison of the distributions of relative amount of TAPSE (**a**), RVFAC (**b**), RIMP (**c**), s′ (**d**), RVLSFW (**e**), RVLS (**f**), respectively. *RV* right ventricle; TAPSE, tricuspid annular plane systolic excursion; RVFAC, RV fractional area change; RIMP, right ventricular index of myocardial performance; s′, tissue Doppler-derived tricuspid lateral annular systolic velocity; RVLS_FW_, RV free wall longitudinal strain; RVLSF, RV longitudinal shortening fraction

Reproducibility of TAPSE, RVFAC, RIMP, s′, RVLS_FW_ and RVLSF in inter- and intra-observers was assessed in all the rats with Bland–Altman analysis. The inter-observer reliability was assessed by using measurements from different observers and then evaluated by examiners blinded to the identity of all groups. The intra-observer reliability over time was investigated by repeating the measurements at monthly intervals (Fig. 4). Despite the rapid heart rates and small heart of rats, the measurements of the conventional parameters and STI parameters still showed high reproducibility in the evaluation of the RV function.

## Discussion

In the current study, we successfully established a MCT-induced PAH model with RV dysfunction and remodeling *in vivo* and comprehensively investigated the effects of E2 treatment on RV dysfunction and myocardial remodeling to illustrate potential mechanisms underlying in PAH. Although this is not the first study to investigate the effect of E2 on RV function in a rat model of PAH, this study comprehensively illustrated the effect focusing on functional changes evaluated by echocardiography. The present study obtained the following findings. Firstly, all rats with MCT injection showed PAH and RVHF, manifested as elevated pulmonary arterial pressure, RVH and decompensated RV function [25, 26]. Significant RV dysfunction was confirmed by the conventional echocardiographic parameters TAPSE, RVFAC, RIMP and s′, which showed similar results as a previous study [26]. Moreover, STI technology was also utilized in this study and showed results consistent with the conventional parameters. We evaluated RVLS_FW_ and RVLSF to further quantify RV function. Secondly, we investigated the effects of E2 on RV morphology and function, which were the key findings in this study. With E2 treatment, RV morphology and function was improved significantly in PAH rats. Further, the parameters of RV structure and function had positive correlations with the serum E2 levels. The significant correlations between the serum E2 levels and hemodynamic (CI), structural (RVH and RV enlargement), and biochemical alterations (BNP) strongly suggest a protective role of E2 in modulating RV function in PAH rats.

### 17β-estradiol inhibited MCT-induced RVH and RV enlargement

Chronic RV pressure overload secondary to PAH resulted in RVH and RV enlargement. Interestingly, our study found that the mid part of the lateral wall of the RV had the most significant increase in diameter. Both RVD/LVD and EI measurements by echocardiography indicated remarkable enlargement of the RV. In contrast, the RV enlargement was dramatically alleviated by E2 treatment. However, the RV maintained a similar shape in rats with E2 treatment compared to that in the control group.

Regarding RVH, one of the most essential parameter was RV wall thickness, which increased significantly in PAH rats, and was maintained in the normal range in E2-treated rats even during pressure overload. For the other parameters, RV/(LV + IVS) was decreased significantly with E2 treatment, as was RV/BW, which was consistent with previous studies [19, 20]. Finally, CSA of RV cardiomyocytes revealed the reversal of RVH with E2 treatment. These findings suggested that estradiol prevented RV cardiomyocyte hypertrophy.

### 17β-estradiol enhanced RV function in PAH

Sex differences have been well documented in diseases of the left ventricle, however, little is known about sex differences in diseases of the RV. Regarding PAH, some studies have indicated that RV function may explain sex differences in the outcome of PAH and estradiol has been associated with better RV function. Recently, several animal studies were conducted in rodents and focused on the effects of E2 on PAH. Although these studies suggested that E2 restored the deteriorated RV structure and function in experimental animal models, the parameters for RV function were not fully evaluated [18, 19].

Our results showed that E2 preserved RV function by maintaining the structure and function of the RV. STI analyses demonstrated that RLVS_FW_ was significantly higher with E2 treatment than without E2 treatment. For regional strain, we found that the strain at the basal portion of the RV was not statistically different from that in the MCT-exposed group and E2 treatment group. This was mainly because of the heterogeneous RV free wall contractility pattern under the increase in afterload, which was presented as a more obvious hypokinesis of the RV apex and mid part of the wall [27]. The strain values of the mid and apical portions were improved significantly with E2 treatment. Combined with the morphologic changes, the myocardium of the mid portion of the RV could be the major improved component of the RV free wall. Regarding the RVLS_FW_, some studies in PAH patients also demonstrated its clinical significance in interpreting sex differences. As suggested in our study, this improvement in RVLS_FW_ in female compared to male patients may at least partly be mediated by the different expression levels and release of endogenous E2 between two genders. RVLSF was another RV functional parameter evaluated in this study. This index has been seldom used in the evaluation of RV function previously. Some published studies showed that it was a useful marker of RV function and was well correlated to the CMR-derived RVEF [15, 28]. In our study, RVLSF also improved remarkably with E2 treatment, further supporting the protective effects of E2 on the RV. Regarding the mechanism of E2 protective actions, the significant reduction in the extent of RV free wall fibrosis may be important. As discussed previously, RV impairment may be due to intrinsic myocardial dysfunction (such as fibrosis) [29]. The structural abnormalities of the RV could also explain the difference in regional strain pattern in PAH rats.

However, inotropic actions of E2 on the RV cannot be fully understood without considering its effects on the pulmonary vasculature. The E2 effects on pulmonary vasculature are controversial, as some published studies showed its beneficial effects such as improving pulmonary arterial remodeling [18, 19], while others demonstrated no beneficial [20], or even detrimental effects [2, 30]. In our study, pulmonary circulation pressure showed no significant alleviation following E2 treatment, which was different from some prior studies. It may be partly explained by the relatively small number of experimental subjects, but also could be related to the highly sophisticated modulatory network regulating E2 mediated effects on the pulmonary vasculature. In this study, although pulmonary vascular resistance (PVR) was not measured directly, it was reasonable to deduce that PVR was higher in MCT-exposed rats. Thus, we also speculated that E2 might have a positive effect on the pulmonary vasculature. Collectively, RV adaptation rather than pulmonary arterial remodeling is the major determinant of enhanced survival in PAH [31, 32].

### Study limitations

Firstly, our study focused on the effects of 17β-estradiol on functional preservation in RV. Caution should be exercised when drawing broader conclusions about other forms of estrogen. In addition, regarding molecular mechanisms that underlies sex differences in PAH, E2 is unlikely to be the exclusive contributor to the preservative effects on RV function. For instance, testosterone may also play a role, although it is indeed beyond the scope of this study. Accordingly, adding a female rat group with ovariectomy would provide more insights into the precise mechanisms. Secondly, considering the effect of RV afterload on RV function, it would be better to evaluate E2 actions on the pulmonary vasculature directly, which may partly explain the effects of E2 on the RV. Moreover, this study only contained a small number of subjects, especially for the MCT-exposed group, where only 5 rats survived. Thirdly, although we comprehensively examined the improvement in RV function in PAH rats with E2 treatment, the molecular mechanisms of these effects warrant further study. Lastly, results obtained in the MCT-induced PAH model may not be necessarily applicable to other types of PAH due to the presence of model and / or practice variations.

## Conclusions

Collectively, 17β-estradiol is as a beneficial modulator of RV function in a rodent model of MCT-induced PAH, indicating that E2 preserves RV myocardial function. These findings at least partly explain the sex differences in PAH.

## Electronic supplementary material

Below is the link to the electronic supplementary material.

Supplementary material 1 (DOC 54.5 kb)
