# Supplementary material for: 17β-estradiol preserves right ventricular function in rats with pulmonary arterial hypertension: an echocardiographic and histochemical study
Source: Int J Cardiovasc Imaging. 2018 Oct 22;35(3):441–50. doi: 10.1007/s10554-018-1468-0 (PMC6454076; doi:10.1007/s10554-018-1468-0)
Supplement: Supplementary file 1 — Supplementary material 1 (DOC 54.5 kb) [file 10554_2018_1468_MOESM1_ESM.doc]

**17β-estradiol Preserves Right Ventricular Function in Rats with Pulmonary Arterial Hypertension: an Echocardiographic and Histochemical Study**

**Supplemental Methods**

**Echocardiographic examinations**

***Evaluation of RV morphology***

The evaluation of RV morphology included the assessment of right ventricular hypertrophy (RVH) and RV enlargement. RV enlargement was demonstrated by two parameters: (1) the ratio of right ventricular diameter (RVD) to left ventricular diameter (LVD) measured at the basal part of both ventricles; and (2) eccentricity index, quantitated as the ratio between LV anteroposterior dimensions and the septolateral dimension, which were measured at the PSAX view according to the guidelines [1]. The parameter for RVH evaluated by echocardiography included RV free wall thickness. The increase of RV free wall thickness indicated RVH, which induced by the increase of pressure overload in this PH model.

***Evaluation of RV function***

***Conventional parameters of RV function.*** The evaluation of RV function was based on the guidelines for the echocardiographic assessment of the right heart in adults [1]. The parameters were obtained by RV-focused apical four-chamber view. TAPSE was acquired in M-mode by measuring from end-diastole to end-systole. RV end-diastolic area (RVEDA) and RV end-systolic area (RVESA) were measured to calculate RVFAC. RIMP was calculated as the ratio of the isovolumic time and the ejecting time, which were measured in the same pulsed tissue Doppler imaging (TDI). The tricuspid s’ was measured by tissue Doppler in the apical four-chamber view (Fig. 3A and B).

***2D-STI analysis.*** Apical four chamber views were specifically optimized to visualize the right ventricle to obtain echocardiographic cine loops by recording ten consecutive heart cycles, with the data stored in a mobile device using the DICOM format. Offline analyses were performed using QLAB 10.3 software (Philips Healthcare).

For the strain analysis, after entering the aCMQ interface to determine the apical four-chamber view and choosing the AP4 option, a region of interest was traced with a point-and-click approach on the endocardium of the RV at end-diastole in the RV-focused view. Then, a larger region of interest was generated and manually adjusted near the epicardium. Special attention was taken to fine-tune the region of interest by using visual assessment during cine loop playback, in order to ensure that the segments were tracked appropriately. After computational analysis, free wall RVLS (RVLS_FW_) was obtained (Fig. 3C and D).

For STI-based assessment of the tricuspid annular motion to evaluate RV longitudinal shortening fraction (LSF), three points were selected in the RV-focused apical four-chamber view as user-defined anatomic landmarks: the insertion of the anterior or septal leaflet into the tricuspid annulus and the RV apex. The software automatically tracked the two points and calculated RV long axis dimension (L) at end-diastole (ED) and end-systole (ES) respectively. RV LSF was defined as (L_ED_ – L_ES_)/L_ED_ (Fig. 3C and D).

***Hemodynamic parameters*** Stroke volume (SV) was evaluated by echocardiography. According to a prior published study [2], pulse Doppler was used to identify maximal velocities within the proximal pulmonary artery and to record it as VTI. Distal RV outflow diameter was measured transversally proximal to the pulmonary valve at end-diastole. SV was determined by using the formula SV = VTI × 3.142 (½ RVOT)^2^, with cardiac output (CO) being calculated as CO = SV × heart rate (HR). To account for estrogen dependent weight changes, cardiac index (CI) was calculated from CO normalized by body weight (BW).

***Immunohistochemistry***

The RV was fixed with 4% paraformaldehyde and, thereafter, embedded in paraffin before being sectioned at a thickness of 4 μm. Then the slides were stained with hematoxylin and eosin (HE) or Masson’s Trichrome. The cross-sectional area (CSA) of cardiomyocytes and the extents of RV fibrosis were determined as previously described [3].

**Supplemental References**

[1] L.G. Rudski, W.W. Lai, J. Afilalo, et al., Guidelines for the echocardiographic assessment of the right heart in adults: a report from the American Society of Echocardiography endorsed by the European Association of Echocardiography, a registered branch of the European Society of Cardiology, and the Canadian Society of Echocardiography, J Am Soc Echocardiogr. 23 (2010) 685-713; quiz 786-688.

[2] A.L. Frump, K.N. Goss, A. Vayl, et al., Estradiol improves right ventricular function in rats with severe angioproliferative pulmonary hypertension: effects of endogenous and exogenous sex hormones, Am J Physiol Lung Cell Mol Physiol. 308 (2015) L873-890.

[3] H.J. Bogaard, R. Natarajan, S. Mizuno, et al., Adrenergic receptor blockade reverses right heart remodeling and dysfunction in pulmonary hypertensive rats, Am J Respir Crit Care Med. 182 (2010) 652-660.
